# Locality and weight sharing shape Hebbian principal-component learning in biologically constrained visual models

**DOI:** 10.3389/fncom.2026.1943064

**Published:** 2026-09-21

**Authors:** Patrick Inoue, Florian Röhrbein, Andreas Knoblauch

**Affiliations:** 1 KEIM Institute (Knowledge Engineering and Information Management), Albstadt-Sigmaringen University, Albstadt, Germany; 2 Department of Computer Science, Chemnitz University of Technology, Chemnitz, Germany

**Keywords:** biological constraints, Hebbian learning, local plasticity, neural computation, receptive fields, visual representation learning, weight sharing

## Abstract

**Introduction:**

Biologically motivated artificial visual learning requires local plasticity rules to be evaluated within architectures whose connectivity assumptions remain explicit. Convolutional Hebbian networks demonstrate that competitive local learning can support visual classification, but their performance is coupled to exact weight sharing, a prior that is not anatomically literal. This study asks whether Hebbian principal component analysis (HPCA) can support representations when spatial locality is preserved but exact translational weight sharing is removed.

**Methods:**

HPCA was evaluated on CIFAR-10 as the primary benchmark, with MNIST and STL-10 used as controls. We compared fully connected HPCA, position-specific locally connected HPCA with fixed receptive fields, non-shared synaptic weights, and population-wise divisive response normalization, and HPCA substitutions in two shared-kernel convolutional Hebbian benchmarks. Representations were learned without supervised feature-layer targets or BP-based feature-layer fine-tuning, frozen, and evaluated through supervised readouts.

**Results:**

Position-specific locally connected HPCA outperformed the fully connected reference. On CIFAR-10, the best locally connected model reached 61.42 ± 0.36%. Shared-kernel HPCA reached 74.51 ± 0.38% in the modular benchmark with common HPCA preprocessing and 76.57 ± 0.55% in the SoftHebb architecture with reference preprocessing.

**Discussion:**

The results show that HPCA remains effective across position-specific locally connected and shared-kernel convolutional architectures, but that performance depends strongly on the surrounding architecture and benchmark protocol. The tested shared-kernel configurations achieved higher accuracy, yet useful frozen visual representations still emerged when exact parameter sharing was removed. Locally connected HPCA therefore provides a controlled model for examining how local plasticity interacts with receptive-field structure and convolutional inductive bias in biologically constrained visual learning.

## 1 Introduction

Deep neural networks (DNNs) have become the dominant computational framework for visual recognition, but their most successful training algorithm, error backpropagation (BP), remains difficult to reconcile with biological learning. BP provides an effective and scalable credit-assignment mechanism by propagating task-level errors through differentiable computational graphs (Werbos, 1974; Rumelhart et al., 1986). This mechanism has enabled high-performing artificial systems across computer vision, natural language processing, and reinforcement learning (Shwartz-Ziv and LeCun, 2024). However, it relies on coordinated forward and backward passes, nonlocal error signals, layer-wise credit assignment, and stored intermediate activations (Lillicrap et al., 2020). This nonlocal credit-assignment mechanism contrasts with biological learning, where synaptic updates are primarily constrained by variables available at or near the synapse (Hebb, 1949).

At the same time, locality alone is not sufficient. Many biologically motivated alternatives to BP remain less competitive on modern visual benchmarks, especially when they avoid supervised feature-layer targets, global error signals, or BP-based fine-tuning. This exposes the central trade-off addressed in this study. A biologically constrained learning rule must reduce the nonlocal coordination required by BP while retaining enough representational capacity to support useful visual discrimination. Biological systems demonstrate that such functional learning is possible under local plasticity constraints, supporting continuous adaptation (Markram et al., 1997; Turrigiano et al., 1998), generalization from limited experience (Tenenbaum, 1998; Lake et al., 2015), and robust behavior under local plasticity and communication constraints (Laughlin et al., 1998; Achard et al., 2006). The relevant question is therefore not whether local learning can match unconstrained supervised optimization in raw accuracy, but how much visual representational structure can be acquired when learning is restricted by locality, spatial connectivity, and the absence of BP-based feature-layer training.

Hebbian plasticity provides a basic class of such local learning rules, with synaptic updates driven by correlations between pre- and postsynaptic activity. However, unconstrained correlation-based updates are unstable and can yield redundant or unbounded representations. Oja's rule and its multiunit extensions address this limitation through stabilizing mechanisms that link Hebbian learning to online principal component extraction and competitive subspace learning (Oja, 1982; Sanger, 1989; Oja, 1989). A distinct information-theoretic formulation links layer-local Hebbian synaptic updates to an information-bottleneck objective, yielding a three-factor rule in which an auxiliary working-memory network supplies a modulatory signal based on information accumulated across sequential samples (Daruwalla and Lipasti, 2024). For visual learning, however, stable synaptic plasticity rules alone do not specify how image structure should be organized across units, receptive fields, and layers. Biological vision exhibits a spatially structured hierarchy, with localized receptive fields in early visual cortex and progressively more selective and tolerant population representations along the ventral stream (Hubel and Wiesel, 1962; Felleman and Van Essen, 1991; Rust and DiCarlo, 2010; DiCarlo et al., 2012). Thus, biologically constrained visual learning requires both local synaptic update rules and architectural structure consistent with the spatial organization of visual processing.

In artificial visual systems, convolutional architectures provide an effective solution to the spatial-structure problem by combining local receptive fields with shared kernels. Local receptive fields restrict each unit to a spatial neighborhood, whereas weight sharing applies the same filter across image locations. Together, these mechanisms improve parameter efficiency, induce translation equivariance at the feature-map level, and support strong recognition performance in large-scale visual tasks (LeCun et al., 1998; Krizhevsky et al., 2012). For biologically motivated models, however, these two mechanisms have different implications. Local receptive fields are consistent with early visual physiology (Hubel and Wiesel, 1962; Fukushima, 1980), whereas exact sharing of synaptic weights across spatial positions is not anatomically literal and is difficult to reconcile with strictly local synaptic plasticity (Pogodin et al., 2021).

Locally connected architectures relax exact weight sharing while preserving spatially restricted receptive fields. They assign position-specific synaptic weights to each unit within a local receptive-field support.^1^ These architectures therefore occupy an intermediate regime between fully connected multilayer perceptrons, which ignore image locality, and convolutional networks, which impose a strong shared-kernel prior. This regime is especially relevant for biologically motivated learning because it allows spatially organized feature extraction to be studied without making exact weight sharing the hidden source of performance. Removing exact weight sharing, however, reduces the parameter efficiency and translational prior that make convolutional architectures effective on difficult image-classification tasks (Pogodin et al., 2021). This creates a trade-off between anatomically motivated position-specific connectivity and the statistical efficiency normally obtained from convolutional weight sharing in translation-invariant image tasks (Lahoti et al., 2024).

Existing biologically inspired visual models address this trade-off in different ways. Some approaches relax exact weight sharing while attempting to preserve convolution-like inductive biases, for example through transformation-rich training of free convolutional networks and locally connected networks (Ott et al., 2020), or through lateral Hebbian mechanisms that induce approximate weight alignment across spatial positions (Pogodin et al., 2021). Other approaches improve Hebbian visual learning by embedding local plasticity rules in shared-kernel convolutional architectures with competition, inhibition, normalization, and supervised readout training (Amato et al., 2019; Miconi, 2021; Lagani et al., 2022; Journé et al., 2023). These Hebbian-CNN models show that local feature learning can operate in stronger visual pipelines, but their performance remains coupled to the shared-kernel convolutional prior.

A further complication is that high accuracy in biologically inspired visual models often relies on additional supervised, contrastive, or gradient-based components. Some approaches use local Hebbian pre-training followed by BP-based fine-tuning, metric-learning objectives, or other supervised adaptation steps. FastHebb improves the computational scalability of Hebbian updates by expressing SWTA- and HPCA-like learning rules in efficient matrix form, but its high-performing use cases combine unsupervised Hebbian feature learning with supervised BP-based fine-tuning (Lagani et al., 2024). Similarly, NM-Hebb combines Hebbian regularization and neuromodulatory consolidation with supervised cross-entropy training and metric-learning fine-tuning (Miličević and Grbić, 2025). Other local-objective methods, including Forward-Forward and Distance-Forward learning, provide important alternatives to end-to-end BP, but rely on label-dependent objectives, positive–negative training phases, or class-conditioned activation evaluations rather than unsupervised feature statistics (Hinton, 2022; Wu et al., 2024). Equilibrium propagation provides a further biologically motivated alternative to conventional BP by training recurrent energy-based networks through contrasts between free and target-nudged equilibrium states. Subsequent work extended the method to deep CNNs (Scellier and Bengio, 2017; Laborieux et al., 2021). It addresses supervised credit assignment through recurrent relaxation, whereas the present HPCA setting learns feedforward features from label-free activity statistics. Taken together, these approaches provide important contextual references, but they differ from the present setting in their objectives, supervision, network dynamics, fine-tuning procedures, or use of BP-based components.

The closest comparison class is therefore formed by Hebbian-CNN models that learn the feature extractor without BP-based feature-layer fine-tuning. The SoftHebb CNN of Journé et al. (2023), grounded in the SoftHebb formulation of Moraitis et al. (2022), provides a strong feedback-free convolutional Hebbian reference, showing that competitive local Hebbian learning can reach high visual-classification accuracy when embedded in an optimized shared-kernel architecture. The modular Hebbian-CNN framework of Nimmo and Mondragón (2025) extends this benchmark line by comparing multiple local learning rules, competition mechanisms, and architectural variants within a common convolutional implementation. These works address the supervised-feature-learning confound more directly than hybrid or local-objective approaches, but they still evaluate local learning under exact convolutional weight sharing.

Exact weight sharing should therefore be treated as an architectural constraint with interpretive consequences, rather than as a neutral implementation detail. It provides a powerful statistical prior for translation-structured image data, but its biological status remains nontrivial because distinct retinotopic populations do not literally share identical synaptic parameters across spatial positions (Pogodin et al., 2021). At the same time, exact weight sharing can be interpreted as an idealized limiting case of functionally aligned receptive-field populations, since transformation-rich visual experience or lateral mechanisms may promote similar filters across spatial positions without explicit parameter tying (Ott et al., 2020; Pogodin et al., 2021). Consequently, performance in convolutional Hebbian models cannot be attributed to the synaptic update rule alone, because the shared-kernel architecture itself supplies a strong inductive bias.

The present study addresses this issue by extending our previous constrained Hebbian formulation (Inoue et al., 2026b) with an explicit population-wise divisive-normalization layer and by evaluating the resulting HPCA feature learner across three architectural regimes. A fully connected multilayer perceptron (MLP) provides a baseline without explicit spatial structure. A position-specific locally connected architecture developed here preserves spatially restricted receptive fields while assigning position-specific synaptic weights. Shared-kernel convolutional regimes then evaluate the same HPCA principle under exact parameter sharing across spatial positions. For this purpose, HPCA was implemented as a dedicated convolutional learning mode in both the modular Hebbian-CNN framework of Nimmo and Mondragón (2025) and the original SoftHebb CNN implementation of Journé et al. (2023). This design separates fully connected, position-specific locally connected, and shared-kernel convolutional architectures as distinct experimental conditions. By treating exact weight sharing as a powerful artificial-network prior and the position-specific locally connected HPCA model as a stricter approximation to retinotopic locality, this design evaluates a shared computational motif across artificial and biologically motivated neural architectures.

In summary, this work makes four contributions. First, it defines a receptive-field-local HPCA update for position-specific locally connected visual learning. Second, it introduces a position-specific locally connected Hebbian architecture combining fixed partially overlapping receptive-field masks, position-specific synaptic weights, and population-wise divisive normalization. Third, it implements HPCA as a shared-kernel convolutional learning mode in the modular Hebbian-CNN framework of Nimmo and Mondragón (2025) and the original SoftHebb CNN implementation of Journé et al. (2023). Fourth, it examines local-rule, architectural, and protocol-dependent effects by comparing fully connected, position-specific locally connected, and two shared-kernel convolutional regimes across CIFAR-10, MNIST, and STL-10 using classification performance and learned receptive-field structure.

## 2 Materials and methods

### 2.1 Hebbian principal component learning

We first define the population-normalized Hebbian update used for the fully connected and locally connected HPCA models. The rule is based on Oja-type neural principal-component learning (Oja, 1989), in which Hebbian potentiation is stabilized by a projection-dependent correction. In the present implementation, competition among hidden units is introduced through population-wise divisive normalization of the postsynaptic responses. The same dense/local update form is used for the fully connected and locally connected variants; the difference between these variants is whether a fixed receptive-field mask is applied to the first-layer synaptic support. The convolutional HPCA substitution is defined separately in Section 2.2.2, because the convolutional benchmark implementations retain their own activation, competition, and normalization mechanisms.

Let **x**∈ℝ^*d*^ denote the presynaptic activity vector of a layer and let **W**∈ℝ^*d*×*m*^ denote the synaptic weight matrix, where *w*_*ij*_ connects presynaptic component *i* to postsynaptic unit *j*. For the fully connected and locally connected HPCA variants, the pre-normalization response of unit *j* is denoted by *h*_*j*_, and the response magnitude entering population-wise divisive normalization is


$$r_{j}=|h_{j}|.$$

The normalized postsynaptic response is then given by


$$z_{j}=\frac{r_{j}}{\sqrt{\beta +\underset{k:\pi \left(k\right)=\pi \left(j\right)}{\sum }r_{k}^{2}}},$$

where β>0 is a stabilizing constant and π(*j*) denotes the postsynaptic response population assigned to unit *j*. Thus, normalization is applied within the population containing unit *j*, rather than globally across the layer.

For a mini-batch, let ${\bar{x}}_{i}$ and ${\bar{z}}_{j}$ denote the batch-averaged presynaptic and normalized postsynaptic activities. Let *s*_*ij*_ denote the structural support of synapse *w*_*ij*_, with *s*_*ij*_ = 1 in the fully connected baseline and *s*_*ij*_ = *m*_*ij*_ in the locally connected variants. The projection term for postsynaptic unit *j* is


$$q_{j}=\overset{d}{\underset{\ell =1}{\sum }}{\bar{x}}_{\ell }s_{\ell j}w_{\ell j}.$$

The implemented Hebbian update is then


$$\begin{array}{l} \Delta w_{ij}=\eta s_{ij}{\bar{x}}_{i}\left({\bar{z}}_{j}-q_{j}\right), \end{array}$$ (1)

where η is the learning rate. In the fully connected baseline, *s*_*ij*_ = 1 for all input–hidden pairs. In the locally connected variants, *s*_*ij*_ = *m*_*ij*_, so updates outside the assigned receptive-field support are suppressed. Within the active synaptic support, the term ${\bar{x}}_{i}{\bar{z}}_{j}$ implements activity-dependent Hebbian potentiation, whereas the projection-dependent term $-{\bar{x}}_{i}q_{j}$ stabilizes the update by reducing growth along the current input-weight projection of unit *j*. Competition among units is introduced through the population-wise divisive normalization of the postsynaptic activities before the update. Equation 1 is the formulation used for the fully connected and locally connected models, whereas the convolutional implementation uses the architecture-adapted formulation defined in Equation 2. In the locally connected model, the structural mask restricts the projection term *q*_*j*_ to the active afferent synapses within the fixed receptive-field support of postsynaptic unit *j*. Because *q*_*j*_ aggregates contributions across multiple afferent synapses of the same unit, the update is receptive-field-local at the circuit level but not strictly synapse-autonomous.

Friston et al. (1993) proposed a computational model for implementing related competitive Hebbian dynamics in biological circuits. In this interpretation, HPCA-type updates combine a synapse-specific Hebbian potentiation term, *x*_*i*_*z*_*j*_, with a projection-dependent stabilizing or decay term that reflects the overlap between presynaptic activity and co-active postsynaptic targets (Lopez et al., 1990; Friston et al., 1993). Thus, synapses are strengthened when presynaptic activity and postsynaptic response co-occur, whereas the corrective term limits unbounded growth. In the present implementation, this principle is instantiated through a population-normalized Oja-type Hebbian update, with competition introduced primarily by population-wise divisive normalization within the response population assigned by π(·). The resulting rule is therefore used as a functional analog of cortical competitive plasticity, without requiring a one-to-one mechanistic identity with any specific biological circuit implementation.

### 2.2 Architectural variants

#### 2.2.1 Fully connected and position-specific locally connected networks

The primary comparison contrasts a fully connected reference model with a locally connected model family, both trained with the Hebbian plasticity rule defined in Equation 1. Within the locally connected family, we evaluate optional energy-pooling and supervised readout variants while keeping the receptive-field-constrained Hebbian feature extractor fixed. Thus, the architectural comparisons separate dense versus receptive-field-constrained synaptic connectivity, the presence or absence of energy pooling, and the structure of the supervised readout.

For the primary fully connected–locally connected comparison, all evaluated settings were matched, including the number of HPCA units, population assignment π(·), preprocessing, augmentation, energy pooling, readout, optimization, training schedule, and seeds. The conditions differed only in feature-layer support, with *s*_*ij*_ = 1 for the fully connected model and *s*_*ij*_ = *m*_*ij*_ for the position-specific locally connected model.

For the fully connected and locally connected HPCA variants, let *x*∈ℝ^*N*^ denote the input vector to the first feature layer. The pre-activation of hidden unit *j* is computed as


$$a_{j}=\underset{i}{\sum }x_{i}s_{ij}w_{ij},$$

where *s*_*ij*_ = 1 in the fully connected baseline and *s*_*ij*_ = *m*_*ij*_ in the locally connected variants. The binary mask *m*_*ij*_∈{0, 1} restricts each locally connected hidden unit to its assigned receptive-field support. The pre-normalization hidden response is


$$h_{j}=a_{j},$$

and the response magnitude used for divisive normalization is *r*_*j*_ = |*h*_*j*_|. Thus, the first-layer response transform in these HPCA variants consists of an absolute response magnitude followed by population-wise divisive normalization, not a learned or separately parameterized activation function. Compared with our previous constrained Hebbian implementation (Inoue et al., 2026b), the proposed divisive-normalization layer replaces the earlier hidden-response normalization and min–max rescaling and restricts normalization to local postsynaptic populations, as proposed in our related work (Inoue et al., 2026a).

In the locally connected variants, the population labels π(*j*) are induced by the receptive-field assignment. In the fully connected baseline, the same population labels are retained for response normalization, but the receptive-field masks are not applied to the synaptic weights.

The fully connected variant imposes no explicit spatial constraint on the synaptic support and therefore serves as a non-spatial reference condition. It retains the same population labels for divisive response normalization, but omits the receptive-field mask during weight initialization and during the manual Hebbian update. Thus, all input components can connect to each hidden unit, while competition remains population-wise rather than layer-global.

In the locally connected variants, this binary mask, collected in the matrix **M**, defines the structural support of the first layer throughout training. The first-layer weight matrix is initialized under the constraint


$$W^{\left(1\right)}\leftarrow M⊙W_{0}^{\left(1\right)},$$

and after each manual Hebbian update the projection


$$W^{\left(1\right)}\leftarrow M⊙W^{\left(1\right)}$$

is reapplied, where ⊙ denotes elementwise multiplication. Weights outside the assigned receptive field are therefore fixed to zero throughout the unsupervised feature-learning phase and do not constitute trainable synapses. In the implementation, the first layer is represented by a dense weight tensor for computational convenience, but the effective trainable connectivity is determined by repeated projection onto the fixed receptive-field support.

After computing the first-layer pre-activation responses, the absolute response magnitudes are divisively normalized using the rule defined in Section 2.1. In the locally connected variants, the population labels π(*j*) are induced by the receptive-field assignment, so competition is restricted to units assigned to the same local population. The normalization is therefore population-based and not determined by receptive-field overlap alone. In the fully connected baseline, the same divisive normalization rule is applied using the population assignment defined for the baseline model. This form of divisive normalization is motivated by canonical normalization models in sensory neuroscience, in which neural responses are scaled by the pooled activity of a local population (Carandini and Heeger, 2012; Heeger, 1992).

The locally connected model family is used to evaluate combinations of energy pooling and supervised readout structure while keeping the receptive-field-constrained Hebbian feature extractor fixed. Energy pooling is included in selected variants after divisive normalization of the first-layer responses. The purpose of this operation is to augment the unit-specific hidden representation with a magnitude-based summary over groups of normalized responses. This operation is conceptually related to classical energy-model formulations, in which squared filter responses are combined to form energy-like responses, and to subspace-norm formulations, in which groups of linear filter responses are represented through pooled magnitude measures (Adelson and Bergen, 1985; Hyvärinen and Hoyer, 2000). In the present architecture, however, energy pooling is used as a deterministic augmentation of the Hebbian representation rather than as a learned pooling mechanism.

In the pooled variants, the normalized hidden response vector **z**∈ℝ^*H*^ is partitioned into contiguous, non-overlapping groups of size *g* along the feature dimension. Let *G*_*r*_ denote the set of indices in group *r*. For each complete group, the pooled energy response is computed as


$$e_{r}=\sqrt{\frac{1}{g}\underset{j\in G_{r}}{\sum }z_{j}^{2}+\epsilon },$$

where ϵ>0 is a numerical stabilizer. This operation corresponds to a root-mean-square energy measure over groups of normalized hidden responses. If *H* is not divisible by *g*, only the largest prefix of **z** containing complete groups is used for the pooled energy responses, while the original vector **z** is retained in full. The pooled energy vector **e** is layer-normalized and concatenated with the original normalized hidden response,


$$\tilde{z}=\left[z,e\right].$$

The resulting representation has dimensionality


$$H+\left\lfloor \frac{H}{g}\right\rfloor .$$

The standard locally connected configuration used a pooling-group size of *g* = 16. To assess sensitivity to this grouping scale, additional conditions were evaluated with *g* = 8 and *g* = 32 while keeping the locally connected HPCA feature extractor, data augmentation, nonlinear readout, and optimization protocol unchanged. These conditions were used as a descriptive hyperparameter-sensitivity analysis rather than as additional inferential comparisons. In variants without energy pooling, the normalized hidden response **z** is passed directly to the supervised stage.

##### 2.2.1.1 Supervised readout training

For the fully connected and locally connected HPCA variants, supervised evaluation is performed only after unsupervised feature learning. The HPCA feature extractor is trained without labels using the local update in Equation 1 and is frozen before supervised evaluation. The supervised stage therefore measures the discriminative utility of the learned representation, rather than modifying the locally learned feature extractor. For the final linear classifier, supervised gradient descent has the local delta-rule form, because the update can be written as a product of presynaptic activity and an output-layer error signal. For squared-error objectives, this corresponds to the classical least-mean-squares or Widrow–Hoff rule (Widrow and Hoff, 1960). For cross-entropy readout training, the same locality structure is retained with the output error defined by the prediction–target residual. This provides a local supervised readout rule for the final classifier without requiring BP through the HPCA feature extractor (Rumelhart et al., 1986; Baldi and Sadowski, 2016).

Some variants use an additional supervised hidden layer before the classifier to test whether the frozen HPCA representation benefits from a more expressive downstream readout. This layer is included only in the nonlinear-readout variants and is optimized by BP for computational efficiency. Because it is applied after HPCA training and does not modify the frozen feature extractor, this optimization is treated as part of the downstream evaluation protocol rather than as part of the unsupervised local feature-learning mechanism. The use of BP in this restricted readout module is therefore not part of the biological feature-learning claim. In principle, the same shallow supervised readout could also be trained by weight perturbation (WP), a biologically plausible perturbation-based estimator that avoids explicit layer-wise error BP by estimating parameter updates from random perturbations and the resulting change in supervised loss (Werfel et al., 2005; Fiete et al., 2007; Züge et al., 2023). Prior work on MNIST showed that WP can reach classification accuracies comparable to BP in shallow networks when sufficient training epochs are used (Stricker et al., 2024). WP is compatible with locality and reward-modulated learning constraints, but perturbation-based estimators scale unfavorably in high-dimensional parameter spaces, especially when perturbations are applied in weight space rather than lower-dimensional activity space (Werfel et al., 2005; Züge et al., 2023). For this reason, BP is used here as the efficient optimizer for the nonlinear-readout variants, while WP provides an in-principle biologically plausible alternative for this restricted shallow readout setting. The central biological claim of the present study remains the locally learned, frozen HPCA feature representation, not the optimizer used for the optional downstream readout.

#### 2.2.2 Shared-kernel convolutional comparison

The locally connected variants described above deliberately omit convolutional weight sharing. To assess whether the same Hebbian PCA principle remains effective under translational weight sharing, we evaluate an architecture-adapted convolutional formulation in two established convolutional Hebbian benchmark settings. The first is the modular Hebbian-CNN benchmark (Nimmo and Mondragón, 2025). The second is the original SoftHebb CNN implementation (Journé et al., 2023). Both comparisons are used as shared-kernel controls rather than as the primary biological model class of the present study.

For these comparisons, we used the official accompanying GitHub repositories of the reference implementations as code bases and extended them by implementing HPCA as an additional convolutional local-learning mode. In the modular Hebbian-CNN framework, this mode was added as an HPCA option in the custom Hebbian convolutional layer, selected in code by setting mode to hpca. In the original SoftHebb CNN implementation, the corresponding HPCA mode was added to the layer-local PyTorch update procedure used for the SoftHebb rule. In both cases, the added mode instantiates the same convolutional HPCA update.

The two PyTorch implementations provide complementary shared-kernel controls. The modular benchmark includes multiple local learning rules, competition mechanisms, and architectural variants, whereas the original SoftHebb implementation provides the corresponding reference architecture and training protocol. The modular benchmark supplies the optimized Hard-WTA BCM reference condition, whereas the original implementation supplies the SoftHebb reference architecture and protocol. The two settings therefore provide complementary shared-kernel controls that differ in local competition, architecture, preprocessing, schedule, and layer-wise hyperparameter configuration.

In each matched HPCA condition, the experimental intervention is the replacement of the original local update rule by the proposed HPCA update. The convolutional architecture, competition mechanism, normalization protocol, supervised readout, optimizer, preprocessing pipeline, and training schedule are kept identical to the corresponding reference implementation. The only implementation-level exception concerns the preprocessing path of the modular Hebbian-CNN benchmark for CIFAR-10 and STL-10. In that reference pipeline, whitening is applied during data loading and training. For the STL-10 experiments, this produced substantial preprocessing overhead on the workstation used for these experiments. We therefore estimated the whitening statistics once, applied the resulting transform to the training and test tensors before model training, and reused the preprocessed tensors in the dataloaders. Training-time augmentation was then applied to the preprocessed training tensors using the same random-crop and horizontal-flip protocol as in the modular benchmark reference configuration. This modification changed only the execution point of the whitening operation in the modular benchmark preprocessing path; it did not change the architecture, learning rule, competition mechanism, feature normalization, readout, optimizer, or training schedule.

For both convolutional implementations, HPCA replaced only the benchmark-defined Hebbian update rule. All benchmark-specific transformations from convolutional preactivations to learning activities, including activation functions, competition mechanisms, normalization, and modulation steps, were retained from the corresponding reference implementation. For a convolutional layer, let **p**_*b*, **r**_ denote the presynaptic input patch extracted from image *b* at spatial position **r**, and let **w**_*j*_ denote the convolutional kernel associated with output channel *j*. We denote the final benchmark-defined activity supplied to the learning rule by ${\tilde{z}}_{b,j,r}$.

The added hpca mode implemented the convolutional Hebbian PCA update


$$\begin{array}{l} \Delta w_{j}\propto \frac{1}{N_{\text{s}}}\underset{b,r}{\sum }{\tilde{z}}_{b,j,r}\left(p_{b,r}-\underset{k}{\sum }{\tilde{z}}_{b,k,r}w_{k}\right), \end{array}$$ (2)

where *N*_bs_ is the number of batch–spatial samples and the index *k* runs over output channels. This update adapts the Hebbian PCA principle to convolutional weight sharing by aggregating the learning statistics across batch and spatial samples. The first term reinforces input patterns correlated with the activity of channel *j*, whereas the second term subtracts the activity-weighted contribution of the output-channel correlation structure. In the implementation, the update tensor is normalized by its maximum absolute value before accumulation in the layer-wise update buffer. Equation 2 therefore defines the controlled learning-rule substitution used in the shared-kernel benchmark comparisons. The shared-kernel convolutional experiments serve as computational control conditions and are not included in the claim of position-specific biological locality, because their updates aggregate statistics across spatial positions and rely on exact kernel sharing.

### 2.3 Datasets and preprocessing

Experiments were conducted on MNIST (LeCun et al., 1998), CIFAR-10 (Krizhevsky, 2009), and STL-10 (Coates et al., 2011). These datasets were selected because they provide a standard progression of visual complexity and because they support direct comparability with the two main convolutional Hebbian benchmark settings considered here, namely the modular Hebbian-CNN benchmark (Nimmo and Mondragón, 2025) and the original SoftHebb CNN benchmark (Journé et al., 2023). MNIST provides a low-complexity grayscale digit-recognition task, CIFAR-10 provides a natural-image benchmark with RGB inputs and larger intra-class variability, and STL-10 further increases image resolution and visual variability. Their use therefore allows the fully connected, position-specific locally connected, and shared-kernel convolutional conditions to be evaluated on overlapping benchmark datasets while preserving comparability with both shared-kernel reference frameworks.

MNIST consists of 70,000 grayscale images of handwritten digits with spatial resolution 28 × 28 pixels. CIFAR-10 consists of 60,000 RGB images of size 32 × 32 pixels. STL-10 consists of RGB images of size 96 × 96 pixels across ten classes. For each dataset, the official training and test splits were used. When a validation set was required for hyperparameter selection, it was drawn from the training split only. Test labels were not used for model selection.

For the fully connected and locally connected HPCA experiments, images were flattened and scaled to [0, 1]. The resulting vectors were processed by a zero-centered and scale-normalized input preprocessing pipeline, consisting of per-sample mean subtraction and RMS normalization, feature-wise standardization using training-set statistics, and final per-sample L2 normalization before the Hebbian PCA update. In the augmented training conditions, augmentations were applied to the preprocessed training vectors, and the resulting samples were re-centered and renormalized to unit Euclidean norm before feature learning. This preprocessing was used to stabilize input offset and scale for Hebbian principal-component updates and is conceptually related to normalization procedures used in local unsupervised visual feature learning (Sanger, 1989; Grinberg et al., 2019; Inoue et al., 2026b).

Data augmentation was treated as an explicit experimental factor in the fully connected and locally connected HPCA experiments. In the augmented conditions, the training data comprised the original preprocessed samples together with flipped, rotated, and spatially shifted variants. These transformations were applied to the training data only, and neither validation nor test data were augmented. For the primary fully connected–locally connected architecture comparison, augmentation status was matched across conditions, so that both models used identical input preprocessing and augmentation protocols. To assess whether the observed architecture-level difference depended on augmentation, the fully connected and locally connected conditions were additionally repeated with data augmentation disabled in both models under the same matched seeds. All remaining preprocessing, feature-learning, and evaluation settings were held constant within this control comparison.

The shared-kernel Hebbian-CNN experiments followed the original preprocessing and augmentation protocols of the respective convolutional benchmark implementations described in Section 2.2.2, unless stated otherwise. This preserves fidelity to the reproduced reference pipelines and ensures that the corresponding HPCA substitutions are evaluated under the same preprocessing, augmentation, normalization, architecture, schedule, and supervised-readout protocol as their respective reference conditions. In the original SoftHebb CNN benchmark, the CIFAR-10 and STL-10 training pipeline uses random cropping with reflective padding and random horizontal flipping, whereas the MNIST augmentation path uses additive Gaussian noise. In the modular Hebbian-CNN benchmark, the main image pipeline uses random horizontal flipping, and, in the ZCA-based condition, application of the corresponding ZCA transformation. ZCA whitening has previously been used in competitive Hebbian convolutional models and was reported to improve performance in related hard-WTA settings (Amato et al., 2019; Nimmo and Mondragón, 2025).

To separate learning-rule effects from preprocessing effects, the convolutional sensitivity analysis additionally evaluates selected reference-rule and HPCA conditions under both the original benchmark preprocessing protocol and the common HPCA preprocessing protocol used for the fully connected and locally connected HPCA experiments. Thus, the reproduced reference results preserve the published preprocessing pipelines, whereas the crossed preprocessing comparisons test how strongly the corresponding convolutional Hebbian benchmarks depend on preprocessing.

### 2.4 Experimental configurations and training protocol

The experimental configurations are organized to compare fully connected, locally connected, and shared-kernel convolutional HPCA conditions, while separately varying energy pooling, input augmentation, dropout, preprocessing, and supervised readout structure where applicable. Complete optimization settings, training schedules, readout hyperparameters, modified source files, and execution commands are provided in the accompanying repository.

The convolutional benchmark conditions are not intended to re-evaluate the full architectural search space of the modular Hebbian-CNN framework (Nimmo and Mondragón, 2025). In particular, the study does not separately compare all architectural variants available in that framework, including the original SoftHebb-style, prior Hebbian-CNN, depthwise-separable, or residual configurations. Instead, the published top-performing Hard-WTA BCM configuration is used as a fixed convolutional reference, and the matched convolutional HPCA condition changes only the local learning-rule mode. This design isolates the effect of replacing the reference Hard-WTA BCM update with the proposed HPCA update under the same shared-kernel convolutional benchmark architecture.

All configurations follow a two-stage training protocol unless explicitly stated otherwise. First, the feature extractor is trained without class labels using the corresponding local Hebbian learning rule. For the fully connected and locally connected HPCA models, this stage updates only the first feature-extraction layer. For the convolutional benchmark models, this stage updates the convolutional Hebbian feature extractor according to the selected learning-rule mode. After unsupervised feature learning, the learned feature-extraction weights are frozen.

Second, a supervised classifier is trained on the frozen representation using the categorical cross-entropy objective. For the fully connected and locally connected variants, the supervised stage consists either of a linear readout or of one additional supervised hidden layer followed by a linear classifier. When dropout is used before the readout in these variants, it is applied only during supervised readout training and not during unsupervised HPCA feature learning. For the convolutional benchmark variants, dropout and supervised-readout regularization follow the corresponding reproduced benchmark protocols (Nimmo and Mondragón, 2025; Journé et al., 2023). Fully connected and locally connected HPCA models are implemented in TensorFlow (Abadi et al., 2016), whereas the shared-kernel convolutional experiments are implemented in PyTorch using the official reference code bases described in Section 2.2.2.

For the parameter-budget control, the locally connected readout was reduced from 2,048 to 208 GELU units such that its total active parameter count approximately matched the 5.9 million parameter scale of the shared-kernel benchmark architectures. The locally connected HPCA feature extractor and all remaining training settings were left unchanged. To quantify architectural scale across the main CIFAR-10 configurations, we report trainable parameter counts in Table 1. For the masked position-specific locally connected layer, only weights within the fixed receptive-field support are counted as active trainable parameters; masked tensor entries fixed at zero are excluded.

**Table 1:** Parameter counts of the evaluated HPCA architectures.

Configuration	Feature depth	Feature parameters	Readout parameters	Total parameters
Fully connected HPCA	1	61.440 M	43.543 M	104.983 M
Position-specific locally connected HPCA	1	1.500 M	43.543 M	45.043 M
Parameter-matched locally connected HPCA, 208-GELU readout	1	1.500 M	4.422 M	5.922 M
Modular shared-kernel HPCA	3	5.647 M	0.246 M	5.893 M
SoftHebb-architecture shared-kernel HPCA	3	5.647 M	0.246 M	5.893 M

Feature parameters correspond to the unsupervised feature-extraction stage, whereas readout parameters comprise the supervised classifier head. The parameter-matched locally connected configuration was chosen to approximate the 5.9 million total-parameter scale of the two shared-kernel architectures. Counts include active feature-layer weights and supervised readout weights and biases; masked zero entries are excluded.

Hyperparameters were selected using validation data drawn from the training split only and were fixed before final test-set evaluation. Test labels were not used for hyperparameter selection, early stopping, or preprocessing decisions. All present-study conditions, including the reproduced reference and protocol-sensitivity conditions, were evaluated over ten independently initialized runs using a fixed set of seeds shared across matched comparisons. Final performance was evaluated on the official test split after supervised readout training. Classification accuracy was used as the primary metric.

### 2.5 Statistical analysis

Statistical inference was performed on seed-level test accuracies. The CIFAR-10 comparison between the fully connected and standard locally connected HPCA configurations was defined as the primary hypothesis test. Because the corresponding configurations were evaluated using matched seeds, differences were analyzed using a two-sided paired-samples *t*-test.

Secondary CIFAR-10 inferential comparisons comprised the standard locally connected configuration versus the no-augmentation, linear-readout, and no-energy-pooling conditions, the optimizer-switch variant versus the standard locally connected configuration, and an augmentation-matched architecture control comparing the locally connected and fully connected conditions with augmentation disabled in both models. Because these comparisons were evaluated using matched seeds, two-sided paired-samples *t*-tests were used. The five secondary CIFAR-10 *p*-values were adjusted using Holm's procedure to control the family-wise error rate.

A separate parameter-budget comparison family comprised the standard versus parameter-matched locally connected conditions and the parameter-matched locally connected condition versus the two shared-kernel HPCA configurations under common HPCA preprocessing. The within-model comparison used a paired-samples *t*-test, whereas the cross-architecture comparisons used Welch's *t*-tests because runs were not naturally paired across implementations. These three *p*-values were Holm-adjusted separately.

For conditions entering inferential analyses, we report *n*, mean, sample standard deviation, and two-sided 95% *t* confidence intervals. Inferential comparisons report the mean difference, its 95% confidence interval, test statistic, degrees of freedom, raw and applicable Holm-adjusted *p*-values, and Cohen's *d*_*z*_ for paired or Hedges' *g* for independent comparisons.

## 3 Results

The results first position the principal HPCA configurations within the CIFAR-10 benchmark context, followed by analyses of protocol sensitivity, locally connected model components, receptive-field structure, and generalization to MNIST and STL-10. Fully supervised CNNs are included only as task-optimized, not mechanistically matched, references.

### 3.1 CIFAR-10 literature context and benchmark positioning

Table 2 summarizes the CIFAR-10 benchmark references and main HPCA conditions used to position the present study. The table focuses on the closest Hebbian-CNN reference conditions and on the present HPCA regimes across fully connected, position-specific locally connected, and shared-kernel convolutional architectures. The supervised SoftHebb CNN architecture is retained as a task-optimized reference for the corresponding shared-kernel architecture.

**Table 2:** Cross-architecture comparisons are descriptive and do not isolate the effects of weight sharing or depth.

Reference	Model	Learning rule	Accuracy [%]
Published references			
Lagani et al. (2022)	Convolutional HPCA, layer-5	Convolutional HPCA	61.12 ± 0.33^a^
Nimmo and Mondragón (2025)	Modular Hebbian-CNN	Hard-WTA BCM	75.20 ± 0.70
Journé et al. (2023)	SoftHebb CNN	SoftHebb	80.31 ± 0.14
Journé et al. (2023)	Supervised SoftHebb CNN architecture	BP	84.0
Main results of this study			
	Fully connected HPCA baseline	HPCA update	52.74 ± 0.59
	Position-specific locally connected HPCA, nonlinear readout	HPCA update	60.56 ± 0.56
	Position-specific locally connected HPCA, nonlinear readout, optimizer-switch variant	HPCA update	61.42 ± 0.36^b^
	Modular Hebbian-CNN, shared-kernel HPCA substitution	HPCA update	70.50 ± 0.59
	Modular Hebbian-CNN, shared-kernel HPCA substitution, common HPCA preprocessing	HPCA update	74.51 ± 0.38
	SoftHebb CNN architecture, shared-kernel HPCA substitution	HPCA update	76.57 ± 0.55

Published rows are shown as reported in the original sources. Present-study rows summarize the position-specific locally connected model and shared-kernel HPCA substitutions.

Literature accuracies are reported as given in the cited publications. Present-study accuracies are reported as mean ± sample standard deviation over *n* = 10 independently initialized runs.^a^For Lagani et al. (2022), the uncertainty denotes the half-width of the reported 95% confidence interval, not a standard deviation.^b^The optimizer-switch variant used the same locally connected HPCA feature extractor and nonlinear readout architecture as the baseline, but changed the supervised readout optimizer from Adam to SGD with momentum after a learning plateau.

Within the Hebbian-CNN reference class, the SoftHebb CNN benchmark provides the strongest published CIFAR-10 result at 80.31 ± 0.14% (Journé et al., 2023). The modular Hebbian-CNN benchmark reports 75.20 ± 0.70% with a Hard-WTA Bienenstock–Cooper–Munro (BCM) rule (Nimmo and Mondragón, 2025), whereas an earlier Hebbian-CNN architecture trained with HPCA reports 61.12 ± 0.33% in the five-layer setting (Lagani et al., 2022). Against this background, the locally connected HPCA condition in the present study achieved 60.56 ± 0.56% with a nonlinear downstream readout. A plateau-triggered optimizer-switch variant of the same locally connected model reached 61.42 ± 0.36%. These results exceeded the fully connected HPCA reference at 52.74 ± 0.59% and reached the same accuracy range as the HPCA-trained Hebbian-CNN architecture (Lagani et al., 2022), despite using position-specific receptive fields without convolutional weight sharing. Formal seed-level comparisons of the fully connected and locally connected conditions are reported in Section 3.3. Numerically, the optimizer-switch variant slightly exceeded the reported five-layer convolutional HPCA reference. At the same time, the stronger modular and SoftHebb CNN benchmarks achieve substantially higher CIFAR-10 accuracy. To assess whether this gap reflects the HPCA update itself or the surrounding shared-kernel benchmark setting, HPCA was substituted into the corresponding Hebbian-CNN pipelines while keeping the remaining configuration fixed.

In the modular Hebbian-CNN benchmark, the HPCA-substituted configuration reached 70.50 ± 0.59% under the original modular benchmark preprocessing and 74.51 ± 0.38% under the common HPCA preprocessing pipeline. The latter condition approached the published modular Hard-WTA BCM reference of 75.20 ± 0.70%, indicating that HPCA performance in this shared-kernel benchmark setting depends strongly on the surrounding preprocessing protocol. In the original SoftHebb CNN architecture, the corresponding HPCA substitution achieved 76.57 ± 0.55% under the original standard-score preprocessing protocol. This result remained below the published SoftHebb CNN reference at 80.31 ± 0.14%, corresponding to a gap of 3.74 percentage points, but exceeded the modular HPCA substitution under both tested preprocessing settings. It also followed the same architecture-dependent ordering as the published shared-kernel references, with the original SoftHebb CNN architecture yielding higher CIFAR-10 accuracy than the modular Hebbian-CNN benchmark (Journé et al., 2023; Nimmo and Mondragón, 2025). Thus, HPCA is not confined to the position-specific locally connected regime, but remains effective when embedded in shared-kernel convolutional Hebbian architectures. The remaining gap to the published SoftHebb reference should therefore not be interpreted as an isolated effect of the HPCA update alone. Rather, the comparison indicates that CIFAR-10 performance in shared-kernel Hebbian models depends on the interaction between the local update rule, convolutional architecture, preprocessing, competition mechanism, learning-rate configuration, schedule, and readout protocol.

To control for total parameter count, we reduced the locally connected readout from 2,048 to 208 GELU units, approximately matching the 5.9 million parameter scale of the shared-kernel architectures. The parameter-matched configuration achieved 59.16 ± 1.52%, compared with 60.56 ± 0.56% for the standard locally connected model, a difference of 1.40 percentage points (*p*_Holm_ = 0.0376). This condition showed markedly higher run-to-run variability than the other locally connected configurations, and the corresponding effect was not confirmed by a Wilcoxon signed-rank test (*p* = 0.084). Under common HPCA preprocessing, the modular and SoftHebb-architecture shared-kernel HPCA configurations achieved 74.51 ± 0.38% and 76.03 ± 0.38%, respectively. They exceeded the parameter-matched locally connected model by 15.35 and 16.87 percentage points, and both Welch comparisons remained significant after Holm correction. Full inferential results are reported in Table 3, and parameter counts are reported in Table 1.

**Table 3:** Seed-level statistical comparisons for CIFAR-10.

Comparison	Δ [pp]	95% CI	Statistic	Raw *p*	*p*_Holm_	Effect size
Locally connected vs. fully connected	7.82	[7.14, 8.49]	*t*_(9)_ = 26.22	8.25 × 10^−10^	—	*d*_*z*_ = 8.29
Standard LC vs. no augmentation	3.67	[3.10, 4.23]	*t*_(9)_ = 14.64	1.39 × 10^−7^	5.56 × 10^−7^	*d*_*z*_ = 4.63
Locally connected vs. fully connected, no augmentation	7.52	[6.99, 8.05]	*t*_(9)_ = 32.34	1.27 × 10^−10^	6.36 × 10^−10^	*d*_*z*_ = 10.23
Standard LC vs. linear readout	3.58	[2.80, 4.36]	*t*_(9)_ = 10.40	2.57 × 10^−6^	7.71 × 10^−6^	*d*_*z*_ = 3.29
Standard LC vs. no energy pooling	0.39	[−0.25, 1.03]	*t*_(9)_ = 1.37	0.202	0.202	*d*_*z*_ = 0.43
Optimizer-switch vs. standard LC	0.86	[0.50, 1.23]	*t*_(9)_ = 5.37	4.53 × 10^−4^	9.06 × 10^−4^	*d*_*z*_ = 1.70
Standard LC vs. parameter-matched LC	1.40	[0.10, 2.69]	*t*_(9)_ = 2.44	0.0376	0.0376	*d*_*z*_ = 0.77
Modular shared-kernel HPCA vs. parameter-matched LC	15.35	[14.25, 16.45]	*t*_(10.14)_ = 31.02	2.22 × 10^−11^	4.45 × 10^−11^	*g* = 13.28
SoftHebb-architecture shared-kernel HPCA vs. parameter-matched LC	16.87	[15.77, 17.97]	*t*_(10.10)_ = 34.13	9.03 × 10^−12^	2.71 × 10^−11^	*g* = 14.62

Positive differences indicate higher accuracy for the first-listed condition. Holm adjustment was applied separately to the five secondary paired comparisons and the three parameter-budget comparisons. The primary fully connected–locally connected comparison was tested separately.

For contextual comparison, broader local-objective methods are considered separately from the mechanistically closer Hebbian-CNN references summarized in Table 2. Forward-Forward with local receptive fields reports approximately 54–59% on CIFAR-10 (Hinton, 2022), whereas Distance-Forward reports 88.20% (Wu et al., 2024). Distance-Forward therefore exceeds the strongest Hebbian-CNN reference in Table 2 by 7.89 percentage points and the supervised SoftHebb CNN architecture by 4.20 percentage points. However, these local-objective methods use label-dependent objectives, positive–negative phases, supervised distance targets, layer-collaborative objectives, or class-conditioned activation evaluations rather than label-free feature extraction from unsupervised synaptic statistics. They are therefore used as contextual performance references, not as mechanistically matched baselines for the present HPCA setting.

Other Hebbian-like CIFAR-10 references outside the convolutional benchmark class provide additional context. In the comparison reported by Ravichandran et al. (2025), several non-convolutional conditions cluster in the low-50% range, including local principal component analysis, local independent component analysis, local random Gabor features, and competitive Hebbian-style models, whereas the feedforward Bayesian Confidence Propagation Neural Network model with Hebbian synaptic and structural plasticity reaches 61.20 ± 0.15% with color-opponent Difference-of-Gaussians preprocessing. This value is in the same numerical range as the locally connected HPCA results reported here. The standard locally connected HPCA condition reached 60.56 ± 0.56%, whereas the plateau-triggered optimizer-switch variant reached 61.42 ± 0.36%, placing the latter numerically slightly above the BCPNN reference. These results place the locally connected HPCA conditions near the upper end of this broader non-convolutional Hebbian-like comparison range. They are included for completeness rather than analyzed as matched baselines, because the corresponding methods combine different plasticity mechanisms, input encodings, preprocessing choices, and architectural assumptions, and are not available as directly comparable shared-kernel convolutional benchmark implementations.

### 3.2 Sensitivity analysis of the convolutional Hebbian benchmark

The preceding comparison indicates that CIFAR-10 performance in convolutional Hebbian networks depends not only on the synaptic update rule, but also on the benchmark protocol in which the rule is embedded. To examine this dependence more directly, we evaluated preprocessing, training duration, learning-rate schedule, and layer-specific learning-rate assignments in the reproduced modular Hebbian-CNN benchmark and the original SoftHebb CNN implementation.

The preprocessing comparison showed a benchmark-dependent effect of input normalization. In the modular Hebbian-CNN benchmark, the HPCA substitution reached 70.50 ± 0.59% under the original modular preprocessing protocol, whereas accuracy increased to 74.51 ± 0.38% under the common HPCA preprocessing pipeline used for the fully connected and locally connected HPCA experiments. This corresponds to an increase of 4.01 percentage points. Thus, in the modular benchmark, the HPCA substitution benefited substantially from the preprocessing protocol used in the present HPCA experiments.

In the original SoftHebb CNN architecture, the HPCA substitution reached 76.03 ± 0.38% under the common HPCA preprocessing pipeline and 76.57 ± 0.55% under the standard-score normalization used in the corresponding reference protocol. This corresponds to an absolute difference of 0.54 percentage points, with the common HPCA preprocessing pipeline yielding lower accuracy in this benchmark setting. Thus, the preprocessing effect differed not only in magnitude but also in direction across the two shared-kernel benchmarks. In the modular Hebbian-CNN benchmark, the common HPCA preprocessing pipeline increased accuracy by 4.01 percentage points, whereas in the original SoftHebb CNN architecture it reduced accuracy by 0.54 percentage points relative to the corresponding reference preprocessing protocol. The preprocessing results therefore do not support a universal benefit of the common HPCA preprocessing pipeline across shared-kernel Hebbian benchmarks. Instead, they indicate that input normalization interacts with the surrounding benchmark setting, including convolutional architecture, competition mechanism, update-rule scaling, learning-rate configuration, schedule, and supervised readout protocol.

The schedule experiments in the original SoftHebb CNN setting further support this broader protocol-dependent interpretation (Journé et al., 2023). In the reproduced SoftHebb reference conditions, using a global learning rate reached 77.17 ± 0.87%, whereas an alternative training schedule reached 79.22 ± 0.80%. Both reproduced conditions remained below the published SoftHebb CNN reference of 80.31 ± 0.14% (Journé et al., 2023). Thus, protocol dependence was not specific to the HPCA substitution. It was also visible in the reproduced SoftHebb reference itself, indicating that performance in this benchmark setting depends on the joint configuration of competition dynamics, layer-wise learning-rate choices, schedule design, and readout optimization.

These results also constrain the interpretation of the difference between the modular Hebbian-CNN benchmark and the original SoftHebb CNN setting. The higher accuracy of the original SoftHebb CNN setting is not explained by training duration alone, although training duration had been discussed as a possible contributor in the modular Hebbian-CNN benchmark (Nimmo and Mondragón, 2025). In the reproduced SoftHebb setting, schedule modifications did not recover the published reference level. Training duration should therefore be interpreted as coupled to the learning-rate schedule and the broader benchmark protocol, rather than as an independent performance factor.

For the modular Hebbian-CNN benchmark (Nimmo and Mondragón, 2025), the dominant tested protocol effect was the preprocessing replacement summarized in Table 4. Additional schedule perturbations produced only small deviations relative to this preprocessing effect and are therefore not reported individually. This does not imply that the modular benchmark is protocol-independent, since it also uses layer-specific learning rates, competition mechanisms, convolutional weight sharing, and a supervised readout protocol comparable in broad structure to the original SoftHebb CNN implementation (Journé et al., 2023). Rather, the observed pattern indicates that the two shared-kernel benchmark settings differ in which protocol components dominate performance. The modular benchmark was most strongly affected by the tested preprocessing replacement, whereas the original SoftHebb CNN setting showed additional sensitivity to the interaction among architecture, soft competition, learning-rate configuration, schedule design, and readout optimization.

**Table 4:** Protocol sensitivity of shared-kernel convolutional Hebbian benchmarks on CIFAR-10.

Model	Protocol condition	*n*	Accuracy [%, mean ±SD]	95% CI
**Modular Hebbian-CNN benchmark** (Nimmo and Mondragón, 2025)				
HPCA substitution	Original modular benchmark preprocessing	10	70.50 ± 0.59	[70.07, 70.92]
HPCA substitution	Common HPCA preprocessing pipeline	10	74.51 ± 0.38	[74.23, 74.78]
**Original SoftHebb CNN architecture** (Journé et al., 2023)				
HPCA substitution	Common HPCA preprocessing pipeline	10	76.03 ± 0.38	[75.76, 76.30]
HPCA substitution	Original standard-score preprocessing	10	76.57 ± 0.55	[76.17, 76.96]
SoftHebb reference	Original preprocessing with global learning rate	10	77.17 ± 0.87	[76.55, 77.79]
SoftHebb reference	Original preprocessing with alternative training schedule	10	79.22 ± 0.80	[78.65, 79.79]

The table reports preprocessing, learning-rate, and schedule perturbations for HPCA substitutions and reproduced reference conditions. Accuracy is reported as mean ± sample standard deviation across independently initialized runs, together with two-sided 95% *t* confidence intervals.

These observations clarify the role of the shared-kernel experiments in the present study. The convolutional HPCA substitutions are not intended as optimized upper bounds for HPCA or as a search for state-of-the-art CIFAR-10 accuracy. They serve as controlled reference conditions for assessing how the convolutional HPCA formulation behaves within shared-kernel benchmark architectures. The observed benchmark-dependent protocol sensitivity further shows that shared-kernel performance reflects the interaction between the local update rule, architecture, preprocessing, competition mechanism, learning-rate configuration, schedule, and readout protocol. Accordingly, the comparison contrasts position-specific receptive-field learning with established shared-kernel benchmark configurations, but it does not independently isolate the effect of exact weight sharing from differences in architectural depth, hierarchy, parameterization, preprocessing, and readout protocol.

### 3.3 CIFAR-10 baseline comparison and position-specific locally connected HPCA ablations

Tables 3, 5 summarize the CIFAR-10 architecture and parameter-budget controls, position-specific locally connected HPCA ablations, pooling-group sensitivity analysis, and the corresponding seed-level inferential comparisons. The analysis first contrasts the locally connected HPCA model family with a fully connected dense-support reference and then evaluates data augmentation, energy pooling, nonlinear supervised readout capacity, supervised readout optimization, and pooling-group size. All complete test statistics, confidence intervals, adjusted *p*-values, and effect sizes are reported in Table 3.

**Table 5:** CIFAR-10 architecture and parameter-budget controls, position-specific locally connected HPCA ablations, and pooling-group sensitivity analyses.

**Condition**	Model	*n*	Accuracy [%, mean ±SD]	95% CI
Fully connected baseline	Fully connected HPCA	10	52.74 ± 0.59	[52.32, 53.17]
Standard locally connected configuration (*g* = 16)	Locally connected HPCA + nonlinear readout	10	60.56 ± 0.56	[60.16, 60.96]
Parameter-matched locally connected configuration	Locally connected HPCA + 208-unit GELU readout	10	59.16 ± 1.52	[58.07, 60.25]
Fully connected, no augmentation	Fully connected HPCA	10	49.37 ± 0.37	[49.11, 49.63]
Locally connected, no augmentation	Locally connected HPCA + nonlinear readout	10	56.89 ± 0.51	[56.53, 57.25]
Optimizer-switch variant	Locally connected HPCA + nonlinear readout	10	61.42 ± 0.36	[61.16, 61.68]
Linear readout only	Locally connected HPCA	10	56.97 ± 0.75	[56.44, 57.51]
No energy pooling	Locally connected HPCA + nonlinear readout	10	60.17 ± 0.94	[59.49, 60.84]
Energy pooling, *g* = 8	Locally connected HPCA + nonlinear readout	10	60.26 ± 0.80	[59.69, 60.83]
Energy pooling, *g* = 32	Locally connected HPCA + nonlinear readout	10	60.13 ± 0.40	[59.85, 60.42]

Accuracy is reported as mean ± sample standard deviation across independently initialized runs, together with two-sided 95% *t* confidence intervals. The standard pooled locally connected configuration uses *g* = 16; the *g* = 8, and *g* = 32 conditions are descriptive sensitivity analyses.

Default locally connected configuration: 20,000 HPCA units; 5 × 5 × 3 receptive fields; η = 5 × 10^−5^; β = 10^−3^; batch size 4,000; 20 HPCA epochs; RMS energy pooling with *g* = 16; 2,048-unit GELU readout; Adam readout training with learning rate 3 × 10^−3^ for 500 epochs. The parameter-matched configuration differs only in readout width (208 GELU units). Full configurations, seed values, and execution commands are provided in the Supplementary material and the accompanying code repository.

Across ten matched runs, the standard locally connected HPCA configuration achieved 60.56 ± 0.56% accuracy, compared with 52.74 ± 0.59% for the fully connected reference, an advantage of 7.82 percentage points (95% CI [7.14, 8.49], *p* = 8.25 × 10^−10^). As implemented here, local connectivity restricts the afferents of each unit to a spatial patch, lowers fan-in, and reduces the number of active parameters. The measured performance gain therefore reflects the combined change and cannot be attributed to spatial locality alone.

A plateau-triggered optimizer-switch variant of the same locally connected model achieved 61.42 ± 0.36%, exceeding the standard configuration by 0.86 percentage points. Both conditions used the same locally connected HPCA feature extractor, data augmentation, energy pooling, and nonlinear supervised readout and differed only in the supervised readout optimization schedule.

The component ablations show that data augmentation and nonlinear readout capacity contributed more strongly than energy pooling to final CIFAR-10 performance. Removing data augmentation reduced locally connected accuracy to 56.89 ± 0.51%, a decrease of 3.67 percentage points relative to the standard configuration. Replacing the nonlinear supervised readout with a linear classifier reduced accuracy to 56.97 ± 0.75%, a decrease of 3.58 percentage points. Thus, both transformation exposure during feature learning and downstream readout capacity affected final classification performance.

To determine whether the architecture-level difference depended on augmentation, the fully connected and locally connected comparison was repeated with augmentation disabled in both conditions. The locally connected model achieved 56.89 ± 0.51%, whereas the fully connected model reached 49.37 ± 0.37%. The locally connected advantage remained 7.52 percentage points (95% CI [6.99, 8.05], $p_{\text{Holm}}=6.36\times 10^{-10}$), close to the 7.82-percentage-point difference observed with augmentation.

Removing the energy-pooling layer produced only a small change, yielding 60.17 ± 0.94% accuracy, corresponding to a difference of only 0.39 percentage points from the standard configuration; the confidence interval for this difference included zero. For the pooling-group sensitivity analysis, mean accuracy was 60.26 ± 0.80% for *g* = 8, 60.56 ± 0.56% for *g* = 16, and 60.13 ± 0.40% for *g* = 32. The means varied by at most 0.43 percentage points, indicating limited sensitivity to the tested pooling configurations.

### 3.4 Receptive-field visualizations

Receptive-field visualizations were used to inspect the features learned during unsupervised local training. This analysis is particularly relevant for the comparison between locally connected HPCA and shared-kernel Hebbian-CNN benchmarks, because the two regimes differ in whether filters are position-specific or shared across spatial locations. The visualized layers differ across regimes for architectural reasons. The locally connected HPCA model contains a single unsupervised HPCA feature-extraction layer, optionally followed by deterministic energy pooling and a supervised readout. Its visualization therefore focuses on this locally learned feature layer. By contrast, the shared-kernel Hebbian-CNN configurations use a multilayer convolutional Hebbian feature extractor, so their visualizations show receptive fields across the three Hebbian convolutional layers used in the best-performing modular benchmark configuration.

Representative receptive fields learned by the locally connected HPCA architecture are shown in Figure 1. The filters were heterogeneous across receptive-field positions and consisted primarily of small-scale chromatic mixtures, local contrast variations, and patch-like high-frequency structure. They did not form a repeated template across the image, consistent with the absence of translational weight sharing. Thus, the locally connected HPCA model learned position-specific local filters rather than a shared bank of reusable convolutional kernels.

**Figure 1:**
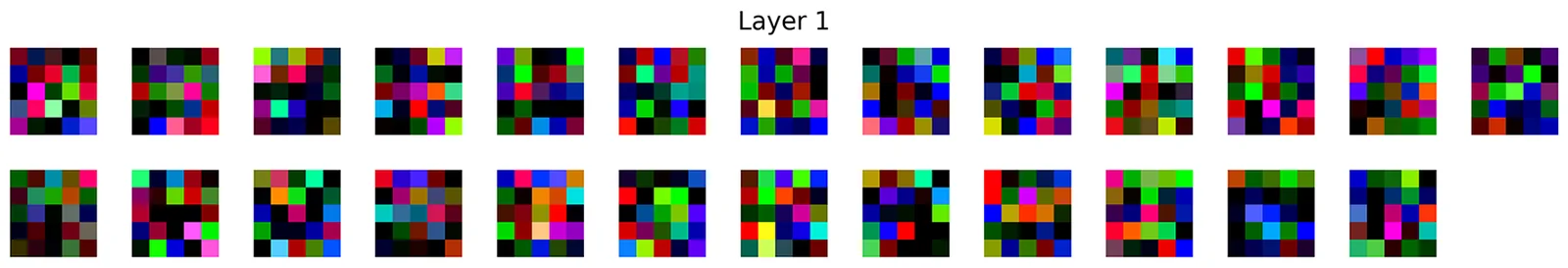
Receptive-field visualizations for the standard position-specific locally connected HPCA configuration on CIFAR-10. The first 25 receptive fields, corresponding to 25 distinct spatial positions, are shown.

The shared-kernel HPCA configurations are shown in Figure 2. In contrast to the locally connected model, the convolutional HPCA architecture exhibited a layer-wise change in receptive-field organization. The first layer was dominated by coarse block-like chromatic and local-contrast filters. The second layer showed more diffuse color-texture combinations with less sharply localized structure. The third layer contained sparse, distributed, high-frequency patterns with scattered color-selective components. This progression indicates that HPCA-based local learning can support structured feature formation both in a position-specific locally connected architecture and in a multilayer shared-kernel convolutional hierarchy.

**Figure 2:**
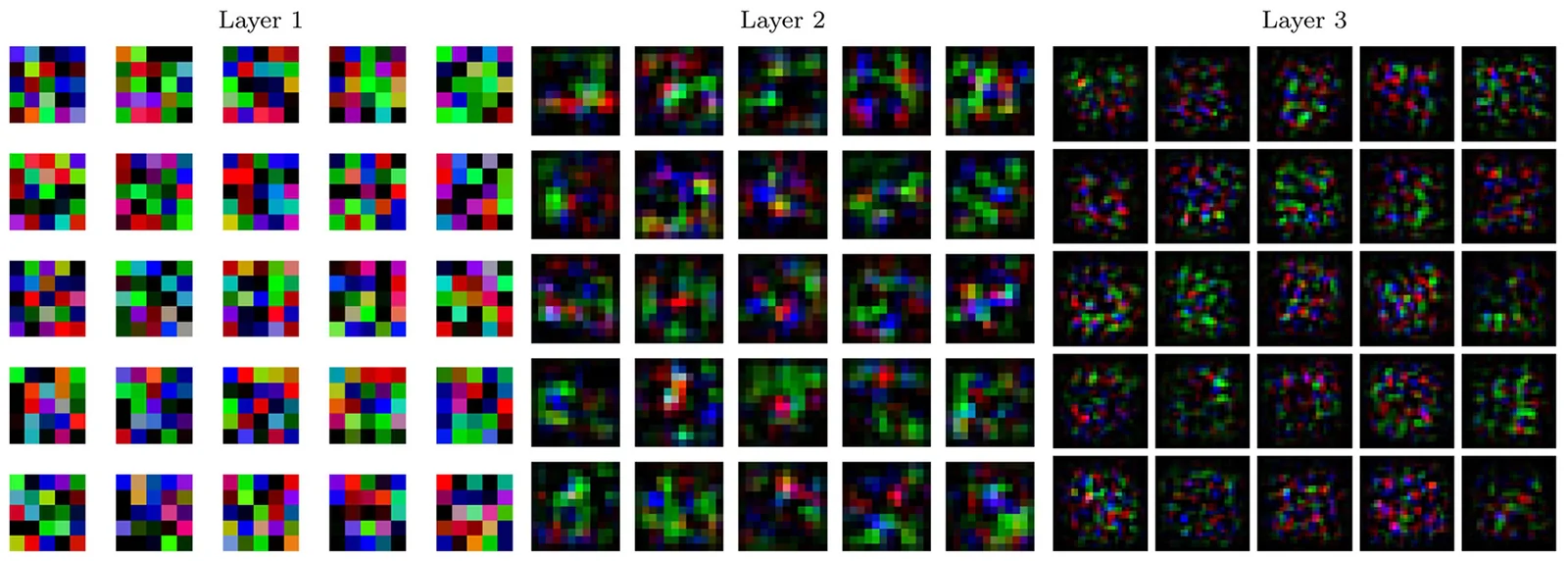
Receptive-field visualizations for the shared-kernel convolutional HPCA configuration on CIFAR-10, obtained by activation maximization. The first 25 of 96 output channels (26.04%) in layer 1, 25 of 384 (6.51%) in layer 2, and 25 of 1536 (1.63%) in layer 3 are shown from left to right.

For context, the reproduced Hard-WTA BCM receptive fields shown in Figure 3 exhibited the qualitative layer-wise organization reported for the modular benchmark, whereas published Soft-WTA and earlier Hard-WTA conditions differed in the sharpness and regularity of edge-, color-, and texture-selective patterns (Nimmo and Mondragón, 2025). The shared-kernel HPCA filters remained within this broader Hebbian range rather than resembling the more regular BP-trained organization. Locally connected HPCA instead produced heterogeneous position-specific first-layer filters. Thus, HPCA supports structured local features, while reusable hierarchical organization is observed in the deeper shared-kernel configurations, where depth and exact parameter sharing co-vary.

**Figure 3:**
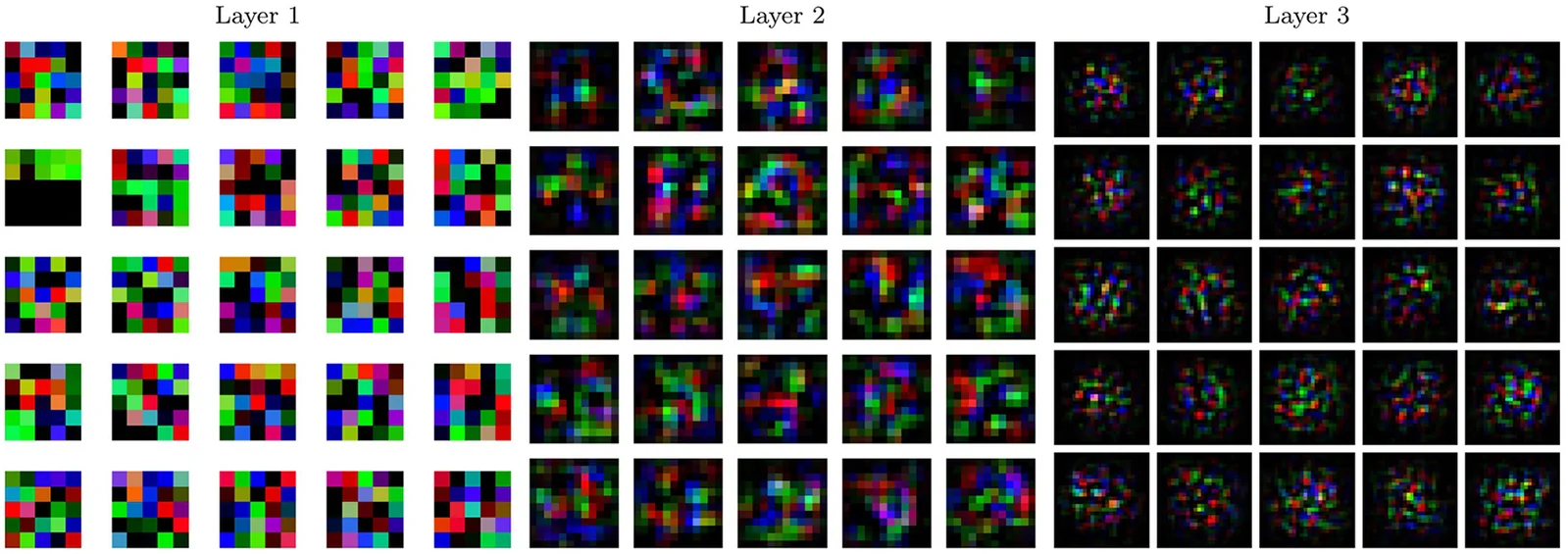
Receptive-field visualizations for the reproduced modular Hard-WTA BCM reference configuration on CIFAR-10, obtained by activation maximization. The first 25 of 96 output channels (26.04%) in layer 1, 25 of 384 (6.51%) in layer 2, and 25 of 1536 (1.63%) in layer 3 are shown from left to right.

### 3.5 Extension to MNIST and STL-10

The principal configurations were additionally evaluated on MNIST and STL-10 to test whether the CIFAR-10 pattern generalized across task complexity. On MNIST, all HPCA conditions reached high accuracy. Across ten runs, the fully connected HPCA baseline achieved 96.98 ± 0.16%, whereas the position-specific locally connected HPCA configuration reached 98.43 ± 0.15%. The shared-kernel HPCA substitutions reached 98.05 ± 0.16% in the modular Hebbian-CNN benchmark setting and 98.71 ± 0.27% in the original SoftHebb CNN architecture. These results are close to the published reference range, with the modular Hebbian-CNN benchmark reporting 98.29 ± 0.22% on MNIST and the SoftHebb CNN benchmark reporting 99.35 ± 0.03% (Nimmo and Mondragón, 2025; Journé et al., 2023). Thus, MNIST does not strongly separate the architectural regimes. The position-specific locally connected condition matched the shared-kernel HPCA substitutions closely, indicating that this low-complexity setting only weakly exposes the advantage of exact convolutional weight sharing.

On STL-10, across ten runs, the fully connected, locally connected, and optimizer-switch HPCA configurations achieved 41.07 ± 0.91%, 49.39 ± 0.47%, and 50.37 ± 0.51%, respectively. The modular and SoftHebb-architecture shared-kernel HPCA configurations reached 66.77 ± 0.61% and 71.40 ± 0.62%, respectively. Both remained below the corresponding published shared-kernel references of 69.49 ± 1.82% and 76.23 ± 0.19% (Nimmo and Mondragón, 2025; Journé et al., 2023). Thus, STL-10 preserved the architecture-dependent ordering observed on CIFAR-10, with a wider gap between the single-layer position-specific model and the deeper shared-kernel configurations.

## 4 Discussion

### 4.1 Main findings and benchmark context

The present results identify HPCA as a circuit-level Hebbian feature-learning rule that remains functional across position-specific locally connected and shared-kernel convolutional architectures. In the position-specific locally connected setting, the complete HPCA configuration substantially outperformed the fully connected reference, yielding a more discriminative frozen representation under spatially structured connectivity. The plateau-triggered optimizer-switch variant further improved the best locally connected CIFAR-10 result. In the shared-kernel convolutional setting, HPCA remained effective when substituted into established Hebbian benchmark architectures, although performance depended strongly on the surrounding architecture, preprocessing protocol, competition mechanism, learning-rate configuration, and training schedule. The cross-dataset results support the same interpretation. HPCA produced highly discriminative representations on MNIST and remained applicable to STL-10, where the higher visual complexity exposed a larger performance gap between the single-layer position-specific locally connected model and the deeper shared-kernel benchmark architectures.

These findings should be interpreted relative to the mechanistic constraints imposed by the present evaluation. The relevant comparison is not absolute parity with high-capacity end-to-end supervised CNNs, which remain stronger task-optimized references, but the ability of a local rule to acquire useful visual representations without supervised feature-layer targets or BP-based feature-layer fine-tuning. Under these criteria, the locally connected HPCA result is meaningful because it is obtained under spatially restricted, position-specific connectivity and without exact translational weight sharing. Among the position-specific locally connected frozen-feature conditions considered here, the best locally connected CIFAR-10 result reached 61.42 ± 0.36%.

Data augmentation was an important contributor to absolute performance in the position-specific locally connected model. Removing augmentation reduced its mean CIFAR-10 accuracy by 3.67 percentage points. Importantly, however, augmentation was matched between the fully connected and locally connected conditions in the primary architecture comparison, and the additional no-augmentation control retained a 7.52-percentage-point locally connected advantage. This value was close to the 7.82-percentage-point difference observed when augmentation was enabled in both conditions. The architecture-level contrast therefore persisted without transformation-based augmentation. In contrast, energy pooling had a comparatively weak influence on CIFAR-10 accuracy under the tested configuration, and accuracy was similarly insensitive to the pooling-group size.

This does not imply superiority over supervised CNNs, shared-kernel Hebbian-CNNs, or local-objective methods that use label-dependent training signals. Rather, it identifies a stricter test case for biologically constrained visual learning. The locally connected HPCA architecture removes exact convolutional weight sharing while retaining spatially restricted receptive fields, local unsupervised feature learning, and frozen-representation evaluation. The resulting CIFAR-10 accuracy is therefore not only a performance result, but evidence that useful visual structure can be acquired without relying on the shared-kernel prior that supports higher-performing convolutional benchmarks. In this sense, the contribution is not that the proposed locally connected HPCA architecture closes the gap to optimized supervised or shared-kernel systems, but that this architecture preserves functional visual representations under position-specific connectivity, where the statistical advantages of exact translational parameter sharing are deliberately removed.

### 4.2 Weight sharing, depth, and protocol dependence in convolutional Hebbian benchmarks

The comparison with Hebbian-CNN benchmarks places exact weight sharing within a broader architectural contrast. The position-specific locally connected HPCA model uses a single unsupervised feature-extraction layer, whereas the shared-kernel benchmark configurations use deeper hierarchical feature extractors. Depth, hierarchy, parameter sharing, preprocessing, competition, and readout protocol therefore co-vary across these configurations and cannot be assigned independent causal contributions from the present experiments. The locally connected HPCA architecture deliberately avoids shared convolutional kernels and instead uses spatially restricted, position-specific synaptic weights. This design does not benefit from parameter reuse across spatial positions, but it provides a stricter abstraction of retinotopic locality because distinct receptive-field positions do not share identical trainable parameters (Hubel and Wiesel, 1962; Fukushima, 1980; Pogodin et al., 2021; Lahoti et al., 2024). The remaining gap to the shared-kernel Hebbian-CNN benchmarks should therefore be interpreted as an empirical difference between the complete evaluated configurations, with exact weight sharing representing one central distinction among several co-varying architectural and protocol factors. The results nevertheless demonstrate that HPCA can acquire useful visual representations without exact weight sharing.

One of these co-varying factors, the number of trainable parameters, can be assessed separately. Reducing the locally connected model to approximately the parameter scale of the shared-kernel benchmarks cost at most about one and a half percentage points of mean accuracy, and at matched size the shared-kernel configurations retained advantages of 15.35–16.87 percentage points under common preprocessing. Because the shared-kernel advantage exceeds the parameter-reduction cost by an order of magnitude, the gap does not arise from differences in total model capacity.

The convolutional HPCA experiments provide a complementary control. When the HPCA update was inserted into established Hebbian-CNN pipelines, it remained competitive with the corresponding Hebbian reference conditions. This shows that HPCA is not intrinsically tied to the position-specific locally connected architecture developed here. At the same time, the comparison between the modular Hebbian-CNN benchmark (Nimmo and Mondragón, 2025) and the original SoftHebb CNN benchmark (Journé et al., 2023) shows that convolutional Hebbian accuracy is strongly shaped by the surrounding architecture and training protocol. The higher raw performance of the original SoftHebb CNN setting should therefore be interpreted as a property of the complete benchmark configuration, including soft competition, exact weight sharing, preprocessing, architectural scale, learning-rate configuration, schedule design, and readout optimization.

This interpretation is important for evaluating the higher classification performance of the original SoftHebb CNN benchmark. The original SoftHebb CNN implementation provides a strong performance reference, but it does not by itself isolate the SoftHebb update rule from the architectural and protocol choices with which it is coupled. The protocol-sensitivity experiments illustrate this point. In the present sensitivity analysis, the modular Hebbian-CNN benchmark showed a pronounced preprocessing benefit from the common HPCA preprocessing pipeline, whereas the original SoftHebb CNN architecture showed a smaller effect in the opposite direction. Additional protocol dependence in the original SoftHebb CNN setting was observed in the reproduced SoftHebb reference conditions, where learning-rate and schedule changes did not recover the published reference accuracy. Input preprocessing, learning-rate configuration, schedule design, competition mechanism, and readout optimization should therefore be treated as parts of the evaluated benchmark configuration, not as neutral operations that can be separated from the learning rule without consequence.

This protocol dependence is also relevant for the biological interpretation of performance differences. A normalization step can improve the stability or performance of a computational learning rule without being a mechanistic model of retinal preprocessing. Biological early vision operates through local, retinotopic, adaptive, cone-dependent, center–surround, and gain-control circuitry under optical, metabolic, wiring, and sampling constraints (Demb and Singer, 2015). Efficient-coding accounts motivate redundancy reduction in early vision (Atick and Redlich, 1992), but whitening or decorrelation alone is not sufficient as a literal account of retinal ganglion-cell receptive-field organization (Graham et al., 2006). The preprocessing and protocol comparisons therefore do not invalidate the corresponding benchmark results. They instead limit the extent to which raw accuracy differences can be interpreted as isolated evidence for the superiority of a particular local plasticity rule.

This interpretation requires one further distinction. Exact convolutional weight sharing is not an anatomically literal assumption, because distinct retinotopic populations do not share identical synaptic parameters across spatial positions (Pogodin et al., 2021). Nevertheless, the shared-kernel regime remains useful as an idealized computational approximation. It can be interpreted as a limiting case of functionally aligned receptive-field populations that are exposed to related visual statistics and governed by similar local plasticity mechanisms. Natural vision exposes retinotopic populations to related local structure across retinal position, scale, viewpoint, and object or observer motion. Such temporally structured visual experience provides a plausible substrate for association-based tolerance learning, consistent with models in which invariance can be learned from transformation sequences and with evidence that unsupervised natural experience can rapidly alter invariant object representations in visual cortex (Földiák, 1991; Li and DiCarlo, 2008). This interpretation is consistent with work showing that transformation-rich training can induce convolution-like behavior when exact parameter sharing is relaxed (Ott et al., 2020), and with proposals that saccade-like experience or lateral Hebbian mechanisms may provide biologically motivated routes toward approximate weight alignment across spatial positions (Pogodin et al., 2021). It is also consistent with the benchmark protocols considered here, in which the Hebbian-CNN pipelines use transformation-based augmentation such as random crops and horizontal flips. These augmentations do not make exact weight sharing anatomically literal, but they operationalize the same computational idea that related visual structure is encountered across spatial positions and transformations during learning. The present results should therefore be read as a comparison between strict position-specific locality and an idealized shared-kernel approximation.

For this reason, the locally connected and shared-kernel experiments support a constrained interpretation of the performance results. HPCA can acquire useful visual representations without exact weight sharing, but the tested shared-kernel configurations provide a strong statistical prior and a substantial performance advantage on the natural-image benchmarks. Conversely, their performance should not be attributed to the local update rule in isolation, because it reflects the joint effects of the learning rule, architecture, preprocessing, competition, and training protocol. The central conclusion is that HPCA remains functional across architectural regimes, while the deeper shared-kernel configurations are associated with more efficient reuse and hierarchical composition of locally learned filters.

### 4.3 Interpreting performance under biological constraints

For the present biological modeling objective, the relevant criterion is constrained performance. The central question is how much visual discrimination can be obtained while preserving mechanisms that remain interpretable as abstractions of biological learning. These include receptive-field-local circuit-level plasticity, spatially restricted receptive fields, position-specific connectivity, limited parameter coordination, implementable stabilization mechanisms, and restricted reliance on labeled feature-layer supervision. This perspective is closer to the optimization problem faced by biological visual systems, which must maintain useful perceptual performance under simultaneous anatomical, metabolic, developmental, and informational constraints.

Under this interpretation, high-performing Hebbian-CNN benchmarks are essential reference systems, but their reported accuracies often reflect more than the local plasticity rule alone. They demonstrate that local learning rules can support strong visual pipelines, while final accuracy also depends on exact weight sharing, preprocessing, competition mechanisms, layer-wise learning-rate schedules, readout design, and other implementation-level choices. The present study therefore evaluates HPCA in a stricter complementary regime, asking whether useful representations can still be obtained under position-specific connectivity without exact shared-kernel parameter tying. The locally connected experiments provide this stricter test, whereas the convolutional substitutions show that the corresponding convolutional HPCA formulation remains effective when embedded in stronger shared-kernel Hebbian-CNN architectures.

### 4.4 Biological interpretation of feature learning and readout

A separate interpretive issue concerns the supervised readout, because the reported accuracies combine unsupervised representation learning with supervised decoding. In this pipeline, class labels are used only after the local HPCA representation has been learned. The HPCA feature extractor is trained from input activity and postsynaptic responses, without supervised feature-layer targets or error BP through the feature extractor. The learned HPCA weights are then fixed before the supervised readout is optimized. Classification accuracy therefore reflects how well the frozen HPCA representation can be decoded, rather than supervised reshaping of the HPCA feature extractor.

For the linear readout condition, supervised optimization is confined to the output mapping from the frozen HPCA representation to class labels. Because the target signal is available directly at the classifier output, the corresponding update can be expressed as a product of presynaptic activity and postsynaptic output error. This is equivalent to the classical least-mean-squares or delta-rule form and does not require hidden-layer error propagation (Widrow and Hoff, 1960; Rumelhart et al., 1986; Baldi and Sadowski, 2016). In this restricted case, the readout is both an evaluation of representational decodability and a local supervised output-layer learning rule.

The nonlinear-readout variants have a narrower interpretation. The additional supervised hidden layer was included to test whether the frozen HPCA representation contains discriminative structure that is not fully exposed by a linear classifier. BP in this module is therefore an optimizer for a downstream decoder, not part of the unsupervised HPCA feature-learning mechanism. It does not update the HPCA weights and does not provide supervised feature-layer targets to the locally learned representation. Consequently, the central biological interpretation remains restricted to the unsupervised HPCA feature extractor and the delta-rule-equivalent output readout, whereas the nonlinear readout is treated as an auxiliary decoding condition.

At the same time, the locally connected HPCA update should not be interpreted as a strictly synapse-autonomous molecular plasticity rule. Its associative component depends on pre- and postsynaptic activity at the individual synapse, whereas the projection-dependent stabilization term requires a neuron-level signal aggregated across the active afferents within the unit's fixed receptive-field support. Population-wise divisive normalization additionally depends on the activity of the assigned local response population. The biological interpretation is therefore restricted to a receptive-field-local circuit-level abstraction, not an isolated-synapse mechanism. Recurrent inhibition, dendritic voltages, and voltage-dependent plasticity provide possible substrates through which neuron- and population-level variables could become available to synaptic learning, but no specific biophysical implementation of the projection term is modeled here (Clopath and Gerstner, 2010; Brendel et al., 2020; Mikulasch et al., 2021).

The locally connected implementation reduces but does not eliminate the abstraction introduced by projection-dependent stabilization and population normalization. In the fully connected reference condition, synaptic support is dense, whereas each locally connected unit receives input only from a fixed receptive field. The associative part of the HPCA update is therefore restricted to spatially local presynaptic activity, moving the model from dense non-spatial learning toward a receptive-field-local circuit-level approximation. The biological claim is therefore not that HPCA constitutes a biophysically detailed model of synaptic plasticity, but that its population-normalized, projection-stabilized Hebbian form provides a controlled circuit-level abstraction of local feature learning under stronger constraints than end-to-end supervised deep learning and biologically inspired approaches that rely on supervised feature-layer targets, BP-based fine-tuning, exact convolutional weight sharing, or label-dependent representation learning.

### 4.5 Limitations and future directions

The present study operates at the level of algorithmic and circuit-level abstraction rather than biophysical implementation. The HPCA update is biologically motivated, but it is not intended as a biophysically detailed model of synaptic plasticity. Its implementation combines projection-dependent stabilization with population-wise divisive normalization, and the present model abstracts away the specific circuit mechanisms that could supply these signals, including recurrent inhibition, dendritic compartmentalization, neuromodulatory gating, temporal spiking dynamics, and ongoing recurrent processing. This limits the level at which HPCA can be interpreted biologically. The relevant claim is therefore not that HPCA provides a complete mechanistic account of synaptic plasticity, but that its population-normalized, projection-stabilized Hebbian form provides a controlled circuit-level abstraction for studying local feature learning under constrained visual architectures.

The experiments also process samples without retaining state across successive inputs. Daruwalla and Lipasti (2024) explicitly link Hebbian synaptic updates to a layer-wise information-bottleneck objective and working memory, using an auxiliary working-memory network to generate a modulatory signal from information accumulated across samples. Incorporating such cross-sample memory into HPCA would require a different learning objective and temporal organization and is not examined in the present study.

Another limitation concerns the nonlinear downstream readout. Replacing it with a linear classifier reduced CIFAR-10 accuracy by 3.58 percentage points, showing that part of the reported performance reflects decoder capacity rather than modification of the frozen HPCA representation. Perturbation-based alternatives such as WP were not evaluated and may be computationally impractical at the present scale. Developing scalable local nonlinear readouts therefore remains a topic for future work (Stricker et al., 2024).

A further boundary of the present study concerns the scope of the convolutional benchmark comparisons. The convolutional HPCA experiments were designed as controlled substitution tests rather than as an exhaustive optimization of Hebbian-CNN architectures. In each benchmark, HPCA was inserted into an established shared-kernel pipeline to test whether an HPCA-based local learning formulation remains effective under exact weight sharing. This design preserves interpretability by keeping the surrounding architecture and training protocol fixed, but it also means that the reported convolutional HPCA results should not be interpreted as optimized upper bounds. Additional tuning of architecture, competition parameters, preprocessing, layer-wise learning rates, or training schedules may further improve performance. The main conclusion is therefore that HPCA remains effective in both the position-specific locally connected regime and the shared-kernel convolutional benchmark regimes, while the absolute performance level remains partly dependent on benchmark-specific architectural and protocol choices.

Future work should address the reduced robustness of position-specific locally connected HPCA. The cross-dataset results indicate that the locally connected architecture remains effective on MNIST and yields useful CIFAR-10 representations, but the gap to shared-kernel Hebbian-CNNs becomes more pronounced on STL-10. This suggests that a single locally connected HPCA feature-extraction layer with optional pooling is not sufficient to recover the hierarchical sample efficiency of convolutional architectures in higher-resolution natural-image settings. Because architectural depth and exact weight sharing were not manipulated independently, the present cross-regime comparisons cannot determine how much of the observed performance gap is attributable to either factor alone. A factorial comparison involving deeper locally connected HPCA models and depth-matched shared-kernel models would be required for such a causal decomposition. A direct next step is therefore to evaluate deeper locally connected HPCA hierarchies, intermediate pooling stages, and lateral interactions between adjacent receptive-field populations. Recent work on Hebbian representation transfer, synapse reuse across datasets, and scalable unsupervised Hebbian representation learning further suggests that staged pre-training or transfer-oriented local learning may improve robustness on more complex visual tasks (Lin et al., 2025; Deng et al., 2025). In a broader scaling direction, global-guided Hebbian learning provides an additional reference for improving Hebbian performance on large-scale tasks, although it relaxes the stricter unsupervised local-learning setting considered here by introducing task-level guidance (Hua et al., 2026). These extensions would test whether position-specific local learning can be made more scalable on complex natural-image datasets without reintroducing exact convolutional weight sharing or replacing the biological learning problem with large-scale BP-based pre-training.

Structural plasticity could extend the locally connected HPCA framework by allowing the receptive-field topology itself to change during learning. In the present model, the receptive-field mask fixes the available synaptic support throughout training, and learning updates only the strengths of existing connections without adding, removing, or reorganizing them. Structural-plasticity models instead allow network connectivity to evolve through the formation, elimination, pruning, or requalification of connections (Knoblauch and Sommer, 2016; Ravichandran et al., 2025; Fahyaz et al., 2026). Extending HPCA to jointly adapt synaptic strengths and receptive-field topology would test whether such mechanisms improve representational efficiency while preserving position-specific connectivity.

Taken together, the results identify HPCA as a circuit-level Hebbian feature-learning rule that remains effective across both position-specific locally connected and shared-kernel convolutional architectures. The central contribution is not a claim of parity with optimized supervised or convolutional systems, but a controlled demonstration of the computational trade-off between functional efficiency and biological constraints. Useful frozen visual representations can still be acquired when feature learning is evaluated under stricter locality, position-specific connectivity, and the absence of exact weight sharing, while the shared-kernel experiments show how the same learning principle behaves when the stronger convolutional prior is restored.

## Data Availability

The original contributions presented in the study are included in the article/Supplementary material. The code used for all experiments, including modified source files, configurations, and execution commands, is also publicly available at https://github.com/PatrickStricker/Hebbian_Learning. No new datasets were generated for this study; the datasets analyzed are the publicly available benchmark datasets MNIST, CIFAR-10, and STL-10, accessed through the official PyTorch torchvision.datasets and TensorFlow/Keras dataset interfaces. Further inquiries can be directed to the corresponding author.

## Footnotes

1 Throughout this article, locally connected refers to architectures with spatially restricted receptive fields and position-specific, non-shared synaptic weights. Shared-kernel convolutional refers to architectures with local receptive fields and exact weight sharing across spatial positions.
