# Supplementary material for: Locality and weight sharing shape Hebbian principal-component learning in biologically constrained visual models
Source: Front Comput Neurosci. 2026 Sep 21;20:1943064. doi: 10.3389/fncom.2026.1943064 (PMC13635126; doi:10.3389/fncom.2026.1943064)
Supplement: Supplementary file 1 [file Data_Sheet_1.zip › Hebbian_Learning-main/softhebb/graphics/cifar10_pre_post_whitening.pdf]

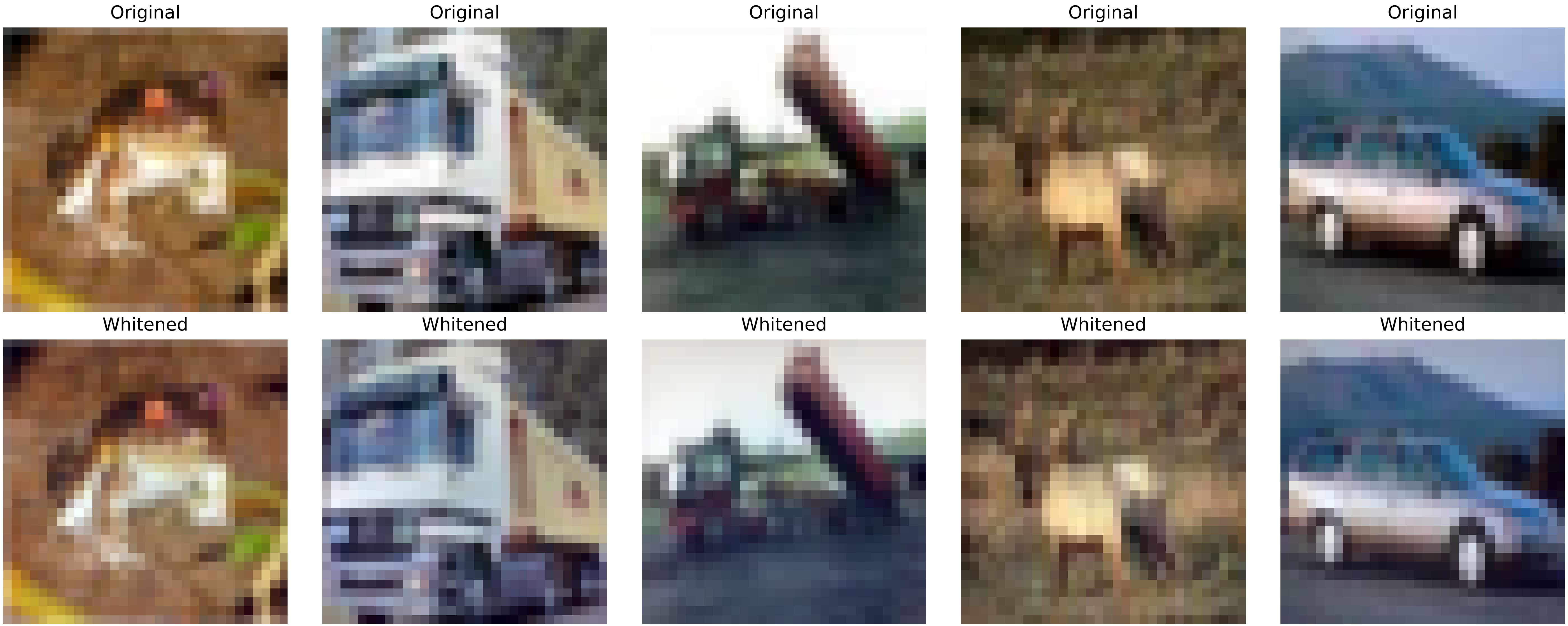
Original
Original
Original
Original
Original
Whitened
Whitened
Whitened
Whitened
Whitened
